# Ontogenetic moulting behavior of the Cambrian oryctocephalid trilobite *Arthricocephalites xinzhaiheensis*

**DOI:** 10.7717/peerj.12217

**Published:** 2021-09-23

**Authors:** Yifan Wang, Jin Peng, Dezhi Wang, Hui Zhang, Xiuchun Luo, Yunbin Shao, Quanyi Sun, Chenchen Ling, Qiujun Wang

**Affiliations:** 1 College of Resources and Environmental Engineering, Key Laboratory of Karst Georesources and Environment, Ministry of Education, Guizhou University, Guiyang, Guizhou Province, China; 2 State Key Laboratory of Palaeobiology and Stratigraphy, Nanjing Institute of Geology and Palaeontology, Chinese Academy of Sciences, Nanjing, Jiangsu, China; 3 School of Geography and Resources, Guizhou Education University, Guiyang, Guizhou, China

**Keywords:** Arthricocephalites xinzhaiheensis, Trilobite, Gradual transition, Ontogenetic moulting behavior, Cambrian, South China

## Abstract

Moulting behaviors in trilobites are a crucial strategy during development. Previous studies have demonstrated inter-and intraspecific variability of moulting behavior in trilobites. Currently, ecdysial motifs for trilobites are considered not stable even within species and fewer detailed studies dealt with moulting behaviors in a single species of trilobite during development. Here a large sample of meraspid to holaspid exuviae of *Arthricocephalites xinzhaiheensis* (131 specimens) from the Cambrian Balang Formation of South China has allowed description of the reasonably complete ontogenic moulting sequence. Both ontogenetic stage and body size reveal gradual transition of configuration from Somersault configuration to Henningsmoen’s configuration during development. Somersault configuration is exclusive till meraspid degree five and exists in subsequent growth stages. This suggests that opening of the facial and rostral sutures allowing the emergence forward of the post-ecdysial trilobite was prevalent in early growth stages. In later development, Henningsmoen’s configuration (showing disarticulation of the cranidium) became more dominant. This study indicates that gradual transition of ontogenetic moulting behavior occurred in oryctocephalid trilobites in the early Cambrian.

## Introduction

The periodic shedding of the old exoskeleton during development is called ecdysis, or moulting, and this is a key feature of Ecdysozoa, which includes three well-supported clades, namely Scalidophora (Loricifera, Priapulida and Kinorhyncha), Nematoida (Nematoda and Nematomorpha) and Panarthropoda (Arthropoda, Tardigrada and Onychophora) (Aguinaldo et al., 1997; Giribet & Edgecombe, 2017; Wang et al., 2019). The major fossil record of moulting comes from the phylum Arthropoda and the literature published on ecdysis in the fossil record mostly concerns the moulting behavior in trilobites (Daley & Drage, 2016). Due to the lack of convincing studies of trilobites preserved in the act of ecdysis (García-Bellido & Collins, 2004; Drage & Daley, 2016; Yang et al., 2019), most work has been based on discarded moult configurations. Henningsmoen (1975) and Drage et al. (2018) named a number of moult configurations and described possible behaviors associated with these. These works contribute to standardizing and better enabling further research into trilobite moulting. Recently, Drage (2019) firstly demonstrated quantitative analysis of trilobite moulting and explored the trends of moulting behavior within Trilobita through the Palaeozoic (Daley & Drage, 2016; Drage, 2019). Previous studies showed that trilobite exoskeleton moulting behaviors are both highly inter-and intraspecifically variable in comparison with what has been observed for other extinct groups (*i.e*., arthropod *Marrella splendens* and fuxianhuiid *Alacaris mirabilis*, scalidophoran worms, lobopodian *Onychodictyon* ) and for modern groups (*i.e.*, green shore crab *Carcinus maenas*, American crayfish *Orconectes limosus*, American lobster *Homarus americanus*, desert locust *Schistocerca gregaria*, priapulid worm *Priapulus caudatus*) (Wang et al., 2019; Daley & Drage, 2016; García-Bellido & Collins, 2004; Yang et al., 2019; Topper et al., 2013; Phlippen et al., 2000; Ayali, 2009; Brandt, 2002). Currently, ecdysial motifs for trilobites are considered not stable even within species and fewer detailed studies dealt with moulting behaviors in a single species of trilobite during development. Lin & Yuan (2009) described a cluster displaying 22 pagetiid individuals at various growth stages. Many specimens were identified as exuviae, which show missing librigenae. Based on these specimens, they considered that Kaili pagetiids have a benthic mode of life after the onset of the meraspid phase (Lin & Yuan, 2009). Morphological change during development affected the style of moulting of phacopine trilobite, which developed fused facial sutures in later ontogenetic stages (Whittington, 1997). Drage, Laibl & Budil (2018) documented the reasonably complete ontogenetic sequence of Dalmanitoidea (Phacopina) and showed there was intraspecific variability in when this occurred. In a recent paper we studied mouting in *Ar. xinzhaiheensis* and found two kinds of moult configurations (Wang et al., 2020). A concentration of well-preserved disarticulated configurations of the early Cambrian oryctocephalid trilobite *Ar. xinzhaiheensis* provides an unparalleled opportunity to allow the distinction between carcasses and exuviae to be made with confidence (Wang et al., 2020), and then explore moulting behavior during development.

Here we describe the complete moulting sequence of *Ar. xinzhaiheensis* from meraspid degree one to the holaspid stage. In early stages, exuviae of *Ar. xinzhaiheensis* exclusively show the Somersault configuration (characterised by an overturned lower cephalic unit), while in later stages Henningsmoen’s configuration (displaying a disarticulated cranidium) became more prevalent. This finding demonstrates that a gradual transition of moulting behaviour occurred in this early Cambrian oryctocephalid trilobite during development.

## Materials & Methods

**Figure 1:**
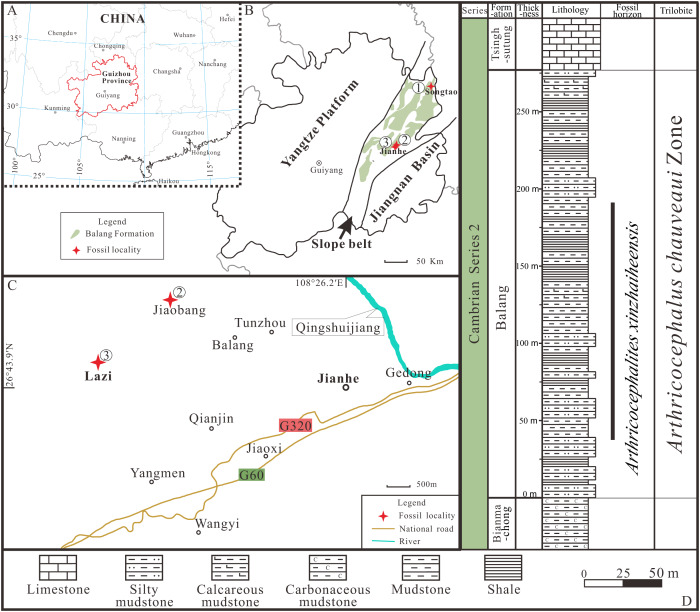
Locality map of the fossil site and stratigraphic horizon of *Arthricocephalites xinzhaiheensis* in Guizhou Province. (A–B) Map of China showing the position of Guizhou Province showing the outcrop distribution of the Balang Formation in grayish green. Fossil localities: ①, Songtao Village (Prefix ST on specimen numbers); ②, Jiaobang Village (Prefix JB); and ③, Lazizhai Section in Lazizhai Village (Prefix JLS). (C) The fossil locality of Jiaobang and Lazizhai Village, Jianhe County, Guizhou Province, South China. (D) Stratigraphical column of the Lazizhai Section, showing the horizon yielding the *Arthricocephalites xinzhaiheensis*. Map modified from Zhou et al. (1979), Peng & Babcock (2001) and Du et al. (2020).

All exuviae of *Ar. xinzhaiheensis* were collected from the Cambrian Balang Formation, Guizhou Province. The Balang Formation crops out in the eastern Guizhou Province and western Hunan Province, south China (Fig. 1). It is also completely located in the Slope Belt between the Yangtze Platform to the present-day west and the Jiangnan Basin to the east (Figs. 1A, 1B) (Zhou et al., 1979; Peng & Babcock, 2001; Du et al., 2020). The formation is a heterogeneous unit but is dominated by greenish-grey shale and calcareous mudstone shale with intercalations of thin-bedded argillaceous carbonates. Abundant fossil assemblages are excavated from the middle to upper part of the Balang Formation at different localities (Peng et al., 2005; Peng et al., 2010; Peng, Zhao & Sun, 2012; Peng et al., 2016; Sun et al., 2014; Shen et al., 2016; Wen et al., 2019; Liu et al., 2018). The biostratigraphic framework of the entire Balang Formation belongs to *Arthricocephalus chauveaui* trilobite Zone dated to the global Cambrian Series 2, Stage 4 (Yan et al., 2014). In this study, the collections are mainly from three localities, including Lazizhai and Jiaobang Village in Jianhe County, Xiangshuidong village in Songtao Country (Figs. 1A, 1B), mostly in Lazizhai section at Lazizhai Village (Figs. 1C, 1D).

**Figure 2:**
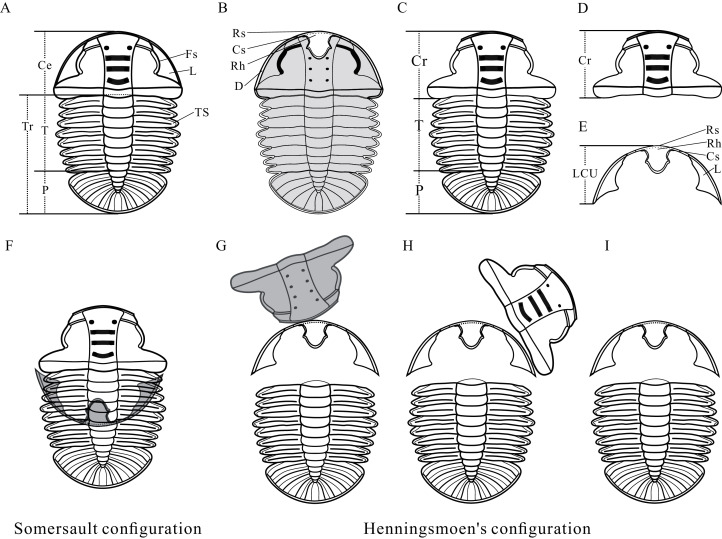
Reconstructions of Arthricocephalites xinzhaiheensis. (A) Dorsal view. (B) Ventral view. (C) Dorsal view of Axial shield. (D) Dorsal view of cranidium. (E) Dorsal view of lower cephalic unit. (F) Somersault configuration. (G–I) Henningsmoen’s configuration. Ce, cephalon; Cr, cranidium; Cs, connective suture; D, doublure; Fs, facial suture; L, librigenae; LCU, lower cephalic unit (all parts of the cephalon except the cranidium); P, pygidium; Rh, rostral‐hypostomal plate; Rs, rostral suture; T, thorax; TS, thoracic segment.

Anatomy of *Ar. xinzhaiheensis* and its moult configurations are described herein (Figs. 2A–2E). The trunk of *Ar. xinzhaiheensis* includes the thorax consisting of a series of articulated segments and the pygidium, which is a posterior group of dorsally conjoined segments. The rostral-hypostomal plate is usually joined to the librigenae as an intact lower cephalic unit (LCU) in *Ar. xinzhaiheensis*. Ontogenetic stages of trilobites have been recognized on the basis of the development of exoskeletal segmentation (Hughes, Minelli & Fusco, 2006; Hughes et al., 2021). The previous work of *Ar. xinzhaiheensis* showed this species had eight thoracic segments in the holaspid stage (Shen et al., 2016). The identified the number of thoracic segments relate specifically to ontogenetic stage (meraspid and holaspid stages) (Hughes et al., 2021; Shen et al., 2016; Fusco, Hong & Hughes, 2014). Here, two parameters were measured in total: (1) sagittal length of the trunk (TrL, see Fusco, Hong & Hughes, 2014); (2) width of the trunk (TrW, width of the first segment). Because of anamorphosis (the increase in the number of trunk segments during ontogeny), we selected the width of the trunk (TrW) as a proxy of general size of *Ar. xinzhaiheensis*. The number of thoracic segements and the width of trunk are interpreted herein as marker of the ontogeny of trilobite. To explore the relationship between moulting behavior and ontogenic stage of *Ar. xinzhaiheensis*, we collected the data of individuals for which it was possible to score the type of moult configurations and to measure size of trunk and the number of thoracic segments.

Here we use an expanded dataset (*n* = 131) to analyse moulting behavior in relation to post-embryonic development. 62 specimens were selected from the previous work (Wang et al., 2020), in addition to 69 new specimens, of which 126 could be measured. A total of five specimens showing the number of thoracic segments have been truncated by rock fracture, so the body size of these specimens wasn’t measured. All specimens presented here are housed in Guizhou Research Centre for Palaeontology, Guizhou University and photographed with Leica M205C stereoscope microscope and Zeiss stereomicro-scope (Model Axio Zoom V16). Measurements were made from images of specimens using the ruler tool of CorelDRAW 2017 (a free trial version).

### Identifying moulting configurations

Numerous criteria have been suggested for identifying fossil moults, based on observations from fossil material in previous studies (Henningsmoen, 1975; Whittington, 1990; Whittington, 1997; Daley & Drage, 2016; Drage et al., 2018; Wang et al., 2020). According to these authors, four important criteria need to be taken into consideration. Criterion 1. Evidence for the opening of exoskeletal sutures, especially cephalic sutures; Criterion 2. A peculiar arrangement of the portions of the exoskeleton, in particular in configurations where certain portions are inverted with respect to the remainder; Criterion 3. Recurring configurations of displaced exoskeletal units; and Criterion 4. Consideration of preservational environment (Wang et al., 2020). The abundance of disarticulated configurations of *Ar. xinzhaiheensis* allowed the distinction between carcasses and exuviae to be made with confidence (Wang et al., 2020). Specimens displaying Somersault and Henningsmoen’s configuration (Figs. 2F–2I) were considered to be slightly disturbed (or undisturbed) exuviae (Wang et al., 2020). Identifying moults of *Ar. xinzhaiheensis* is discussed briefly below.

Somersault configuration involving inversion of the LCU is highly unlikely to have been produced by chance. The specimens in Henningsmoen’s configuration consist of LCU and trunk, and the cranidia are inverted, rotated or missing. As Whittington (1997) noted, it is difficult to accept that burial could take place without some disturbance of the remains (Wang et al., 2020). The positioning of the LCU and missing cranidium imply that the specimens suffered slight disturbance. In this study, two configurations of *Ar. xinzhaiheensis* meet criteria 1–4 and these arrangements are interpreted as resulting from a slightly disturbed (or undisturbed) exuvia which are consistent with Wang et al. (2020).

## Results

### Fossil description

**Figure 3:**
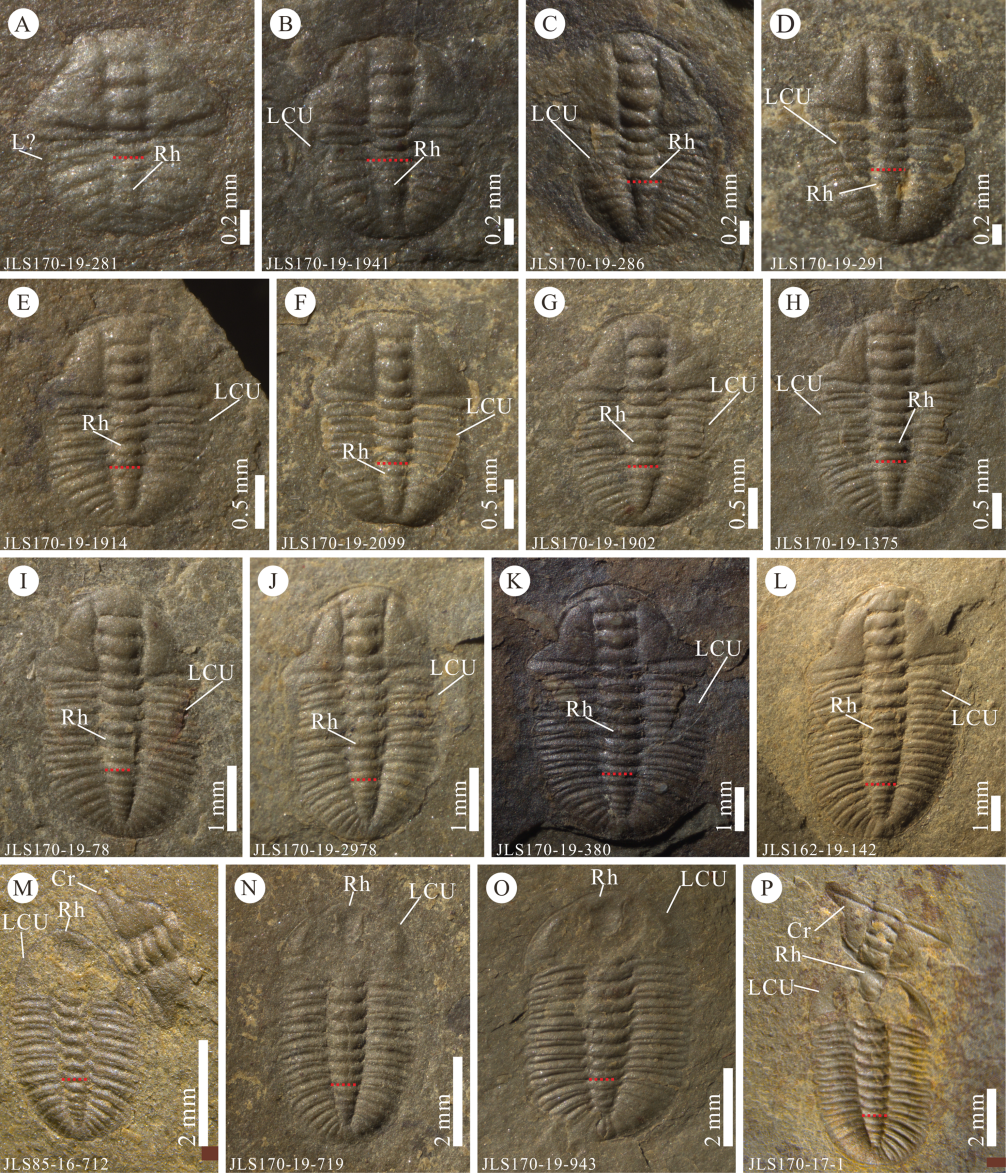
Examples of Somersault configuration and Henningsmoen’s configuration in *Arthricocephalites xinzhaiheensis* from the Cambrian Balang Formation, Guizhou Province, South China. (A–L) Inverted lower cephalic unit; (M–P) disarticulated cranidium. (M) and (P) previously illustrated by Wang et al., 2020, (D) and (A), respectively; the red dashed line indicates the thoracic/pygidial boundary. A meraspid degree 1; (B) meraspid degree 2; (C, D) meraspid degree 3; (E, F) meraspid degree 4; (G, H) meraspid degree 5; (I, M) meraspid degree 6; (J, K, N) meraspid degree 7; (L–P) holaspid stage. Abbreviations: L?, librigenae is not clear; LCU, lower cephalic unit (all parts of the cephalon except the cranidium) (see Fig. 2 and Table S1 for list of abbreviations and details). Figure © 2020 Lethaia Foundation.

Specimens of *Ar. xinzhaiheensis* range in our sample 0.52–9.34 mm in sagittal trunk length and 0.94–7.87 mm in transverse trunk width (Table S1). According to the morphological characteristics of specimens and previous work (Wang et al., 2020; Shen et al., 2016), the number of thoracic segments and corresponding developmental stage can be confirmed (Fig. 3). Specimens in Somersault configuration (*n* = 49) include individuals from meraspid degree one to the holaspid stage (Figs. 3A–3L), while specimens in Henningsmoen’s configuration (*n* = 82) include individuals from meraspid degree six to the holaspid stage (Figs. 3M–3P). Figures 3B–3L, examples of Somersault configuration, show the inverted LCU lying beneath the thorax, confirming that the LCU must have been inverted anteriorly during moulting. As these specimens are internal moulds, the underlapping LCU impressed upon the overlying trunk is a major characteristic for the recognition of Somersault configuration in *Ar. xinzhaiheensis*. Apart from this, inverted librigenae extending from two sides of the trunk and broken thoracic segements revealing part of the inverted LCU are also important signals for identification. The specimen in Fig. 3A is also considered as Somersault configuration based on the inverted rostral-hypostomal plate lying beneath the trunk though the inverted librigenae is not very clear. Specimens of Henningsmoen’s configuration display disarticulated cranidia (invertion, lateral rotation or missing) and the joined LCU, which was held in place *via* integument, remains largely *in situ* with respect to the trunk (Figs. 3M–3P). As such these specimens are generally easily recognizable as moults belonging to Henningsmoen’s configuration. This configuration showing inverted or rotated cranidia in close association preserves substantial information of moulting. Those with missing cranidia are more likely caused by slight disturbance (Whittington, 1997; Wang et al., 2020), including the possibility of being carried away by the freshly moulted trilobite.

### Analysis of dataset

**Figure 4:**
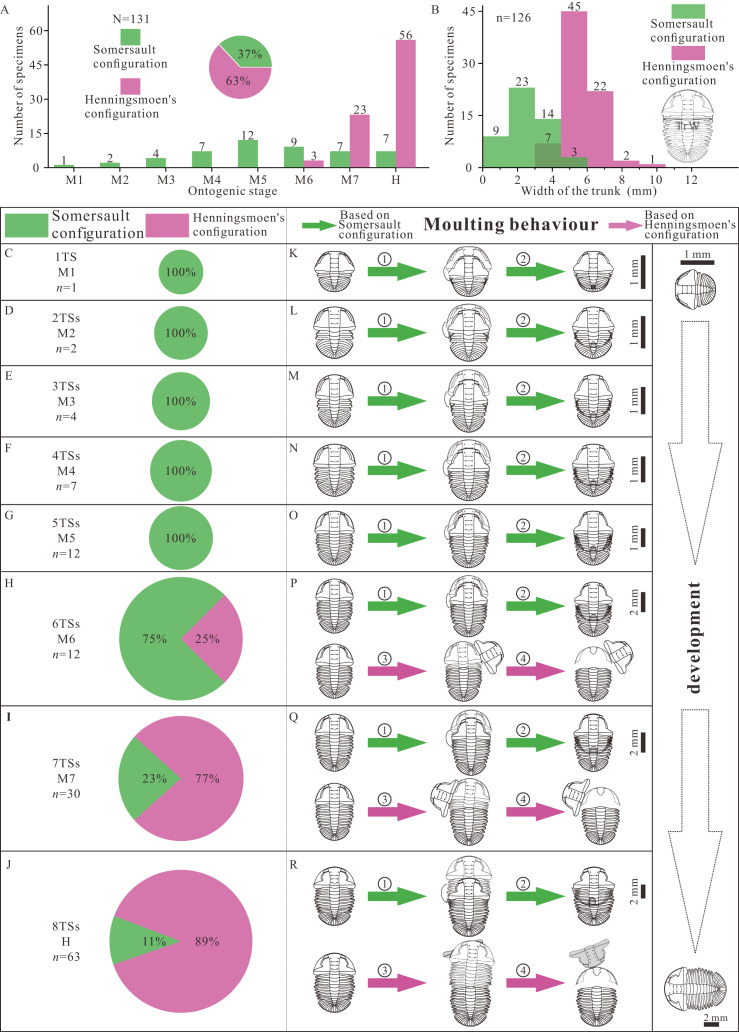
Result and reconstructions showing gradual transition of moulting behavior in *Arthricocephalites xinzhaiheensis* during development. (A) Number of specimens in different growth stages. Pie chart showing different proportions of moulting configurations; (B) Size distribution of trunk length. The unit is [0, 1.5). (C–J) Pie chart showing different proportions of moulting configuration in different growth stages; (K–R) Suggested moulting behavior in *Arthricocephalites xinzhaiheensis* during development. N, total specimens; n, measured specimens; *n*, number of specimens in different growth stages. Abbreviations: TrW, the width of the trunk (width of the first segment); 1TS, 1 thoracic segment; 2-8TSs, 2–8 thoracic segments; M1-7, meraspid degree 1–7; H, holaspid stage. ①, facial and rostral sutures opened; ②, cephalothoracic joint opened, facial and rostral sutures opened, disarticulated cranidia inverted forward or rotated laterally; ③, newly moulted trilobite egressing from old exuviae. (see Fig. 2 and Table S1 for details).

There are two groups of data, the number of thoracic segments and the size of trunk, which are key markers of the developmental phase of the trilobite. The measured specimens show a reasonably complete ontogenetic sequence. The composition of moult configuration in different growth stages is significantly influenced by development (Figs. 4A, 4B). There is gradual transition from Somersault to Henningsmoen’s configuration during development, both with reference to ontogenetic stage (Fig. 4A) and to body size (Fig. 4B). Somersault configuration accounts for 37% of all specimens which appear in all stages of *Ar. xinzhaiheensis*, while nearly two thirds of specimens display Henningsmoen’s configuration which only exists in meraspid degree six, meraspid degree seven and holaspid stage (Fig. 4A). Somersault configuration is exclusive till meraspid degree five and the proportion gradually decrease from meraspid degree six (Figs. 4A, 4C–4J). In later development, Henningsmoen’s configuration gradually becomes dominant with the development of individuals (Figs. 4A, 4C–4J).

## Discussion

### Ontogenetic moulting behavior of *Arthricocephalites xinzhaiheensis*

The reasonably complete ontogenetic sequence of moulting configurations provides an unparalleled opportunity to discuss the developmental moulting behavior of this trilobite. Somersault configuration and Henningsmoen’s configuration represent different moulting processes (Drage et al., 2018; Wang et al., 2020; Whittington, 1990; Chen, Han & Zhao, 2008). Based on previous studies, the sequence of events based on Somersault configuration is summarized here (Figs. 4K–4R, ①, ②). The trilobite began a relative severe dorsal flexure. Then opening of the cephalic sutures (facial and rostral sutures) would have allowed the emergence forward of the post-ecdysical animal. As the animal continued to withdraw from the old cuticle, the LCU tilted up vertically and inverted. Meanwhile, the process which may have led to formation of Henningsmoen’s configuration can be presumed as follows. The trilobite began an initial downward (towards the sediment) angling of the cephalon and upwards flexure of the dorsal thorax producing a partial enrollment (Figs. 4P–4R, ③, ④). This high angle of cephalon resting on the sediment led to the cephalothoracic joint to break. The facial and rostral sutures successively opened. Subsequently, the disarticulated cranidia which provided wide exuvial gape were inverted forward or rotated beside when the newly moulted trilobite anteriorly egressed the old exoskeleton. Finally, the old exoskeleton was shed and the LCU which connected to the articulated trunk by integument preserved in suit. The composition of moult configurations (Figs. 4A–4J) suggested gradual transition of moulting behavior in *Ar. xinzhaiheensis* during development. Here we summarise the ontogenetic moulting behavior of *Ar. xinzhaiheensis* (Figs. 4K–4R). Somersault configuration is exclusive till meraspid degree five (Figs. 4K–4O) and exists in subsequent growth stage. This suggests that opening of facial and rostral sutures allowing the emergence forward of the post-ecdysial trilobite is prevalent in early growth stage. With the subsequent development of individuals, the composition of Henningsmoen’s configuration is increased. This demonstrates that the operations of the cephalic sutures (facial sutures and rostral suture) and cephalothoracic joint for newly moulted trilobite gradually become dominant behavior in later growth stage (Figs. 4H–4J, 4P–4R).

The change from Somersault to Henningsmoen’s configuration in *Ar. xinzhaiheensis* does not imply a change in the importance of the cephalic sutures. The operation of the cephalic sutures in small specimens generally seems to have been adequate, whereas in larger specimens both the cephalic sutures and disarticulation of the cephalothoracic joint were required. Essentially what we are seeing is that in the Somersault configuration the ecdysial gape is opened by a downwards movement of the LCU relative to the cranidium, whereas with Henningsmoen’s configuration the LCU stays in place while the ecydisal gape is opened by the removal of the cranidium. This implies more dorsal flexure in small specimens (resulting in the tipping over of the LCU) and less flexure in larger specimens (at least when they are extracting themselves from the exuviae). Actually it is difficult to say why there was a change in moulting strategy during development in this study. Perhaps small specimens could enroll more efficiently than larger specimens (*e.g.*, for protection). For example, in *Estaingia bilobata* from the Emu Bay Shale meraspides are often found enrolled, whereas this is less common in larger specimens (Holmes, Paterson & García-Bellido, 2021).

### New insight of variability in trilobite mouting behavior

Compared with a signature ecdysial pattern in extant macrobenthic marine arthropods (crabs, shrimp, lobsters, horseshoe crabs), moulting behavior in trilobites reveals obvious variation (Drage, 2019; Phlippen et al., 2000; Ayali, 2009; Brandt, 2002; Drage et al., 2019; Zong, 2020). Brandt (2002) considered that trilobites seem to have been opportunistic moulters by discussing ecdysial efficiency and evolutionary efficacy among marine arthropods. The most common moulting behavior for trilobites is utilizing the cephalic sutures, as expected by the almost ubiquitous presence of librigenae with facial sutures in trilobite morphology (Drage, 2019). Devonian phacopids possessed fused facial sutures in holaspides, displaying the inverted complete cephalon (excluding the hypostome) in exuviae (Henningsmoen, 1975; Whittington, 1997; Zong & Gong, 2017; Rustán et al., 2011). Drage, Laibl & Budil (2018) considered that facial suture fusion in Dalmanitina (phacopid trilobites) was intraspecifically variable which necessitate unstable alterations to exoskeletal moulting behavior. These works lead a realization that ecdysial motifs for trilobites are not stable within species. Previous studies also indicated, however, that the same moulting behavior exists in different trilobite clades and intraspecific variation of this strategy also appears in some trilobites (Drage et al., 2018; Drage, 2019; Wang et al., 2020; Whittington, 1990; Chen, Han & Zhao, 2008; Zong & Gong, 2017; McNamara, 1986; McNamara & Rudkin, 1984; Speyer, 1985; Zong, 2020). Some disarticulated carcasses were considered as disturbed exuviae in some studies (Drage et al., 2018; Whittington, 1990; McNamara, 1986). Abundant exuviae of *Ar. xinzhaiheensis* show that variation in moult configurations is regular in this species, implying a gradual transition of moulting behaviour across ontogeny.

## Conclusions

A concentration of well-preserved exuviae of the oryctocephalid trilobite *Ar. xinzhaiheensis* from the Cambrian Balang Formation, South China presented two types: Somersault configuration which involves inversion of the lower cephalic unit, and Henningsmoen’s configuration which displays a disarticulated cranidium, reflecting different moulting processes. Both ontogenetic stage and body size of specimens reveal a reasonably complete ontogenetic sequence and indicate gradual transition from Somersault configuration to Henningsmoen’s configuration during development. Somersault configuration is exclusive till meraspid degree five and exists in subsequent growth stage. This suggests that opening of facial and rostral sutures allowing the emergence forward of the post-ecdysial trilobite was prevalent in early growth stages. With the subsequent development of individuals, the disarticulated cranidium of Henningsmoen’s configuration gradually became dominant. Compared with a signature ecdysial pattern in extant macrobenthic marine arthropods, moulting behavior in trilobites shows obvious variation. This study indicates that variability of moulting behavior in this early Cambrian trilobite was regular, showing a gradual transition of moulting behaviour across ontogeny.

## Supplemental Information

10.7717/peerj.12217/supp-1 Supplemental Information 1 Moulting configurations in *Arthricocephalites xinzhaiheensis*. Prefix: JLS = Lazizhai Section, Jianhe Country, Guizhou province, South China; JB =Jiaobang Village, Jianhe County, Gui zhou province, South China; ST=Songtao Country, Gui zhou province, South China; M1-7 = Meraspid degree 1–7; H = holaspid stage; / = no data (the trunk is broken); 1 = Yes; 0 = No; ★ = data sources come from Wang et al. (2020); ★★ = data sources come from Wang et al. 2020 and the number of thoracic segment is revised; JLS170-16-1 and JLS160-19-163 appearing Wang et al. (2020) are excluded here. These specimens were measured by the ruler tool of CorelDRAW 2017 (a free trial version).

## Additional Information and Declarations

### Data Availability

The following information was supplied regarding data availability: The raw measurements are available in the Supplementary Table.
