# Supplementary material for: Ontogenetic moulting behavior of the Cambrian oryctocephalid trilobite Arthricocephalites xinzhaiheensis
Source: PeerJ. 2021 Sep 23;9:e12217. doi: 10.7717/peerj.12217 (PMC8465991; doi:10.7717/peerj.12217)
Supplement: Supplemental Information 1 — Prefix: JLS = Lazizhai Section, Jianhe Country, Guizhou province, South China; JB =Jiaobang Village, Jianhe County, Gui zhou province, South China; ST=Songtao Country, Gui zhou province, South China; M1-7 = Meraspid degree 1–7; H = holaspid stage; / = no data (the trunk is broken); 1 = Yes; 0 = No; ★ = data sources come from Wang et al. (2020); ★★ = data sources come from Wang et al. 2020 and the number of thoracic segment is revised; JLS170-16-1 and JLS160-19-163 appearing Wang et al. (2020) are excluded here. These specimens were measured by the ruler tool of CorelDRAW 2017 (a free trial version). [file peerj-09-12217-s001.docx]

Table S1. Moulting configurations in *Arthricocephalites xinzhaiheensis*. Prefix: JLS= Lazizhai Section, Jianhe Country, Guizhou province, South China; JB=Jiaobang Village, Jianhe County, Gui zhou province, South China; ST=Songtao Country, Gui zhou province, South China; M1-7= Meraspid degree 1-7; H= holaspid stage; / = no data (the trunk is broken); 1= Yes; 0= No; ★= data sources come from *Wang et al. (2020)*; ★★= data sources come from *Wang et a*l. *2020* and the number of thoracic segment is revised; JLS170-16-1 and JLS160-19-163 appearing *Wang* *et al*. *(2020)* are excluded here.

| **Moulting configurations in *Arthricocephalites xinzhaiheensis*** | | | | | | | |
| --- | --- | --- | --- | --- | --- | --- | --- |
| Serial number | **Somersault configuration in *Arthricocephalites xinzhaiheensis*** | | | | | | |
|  | Number | Number of thoracic segment | Stage | Length of trunk (mm) | Width of trunk (mm) | Inversion of LCU | Data sources |
| 1 | JLS170-19-281 | 1 | M1 | 0.52 | 0.94 | 1 | This study |
| 2 | JLS170-19-1941 | 2 | M2 | 0.84 | 1.16 | 1 | This study |
| 3 | JLS170-19-352 | 2 | M2 | 0.85 | 1.21 | 1 | This study |
| 4 | JLS170-19-286 | 3 | M3 | 0.87 | 1.16 | 1 | This study |
| 5 | JLS170-19-720 | 3 | M3 | 0.92 | 1.15 | 1 | This study |
| 6 | JLS170-19-2952 | 3 | M3 | 1.03 | 1.49 | 1 | This study |
| 7 | JLS170-19-291 | 3 | M3 | 1.13 | 1.49 | 1 | This study |
| 8 | JLS170-19-1914 | 4 | M4 | 1.02 | 1.42 | 1 | This study |
| 9 | JLS170-19-2099 | 4 | M4 | 1.15 | 1.53 | 1 | This study |
| 10 | JLS84-19-120 | 4 | M4 | 1.18 | 1.33 | 1 | This study |
| 11 | JLS170-19-1151 | 4 | M4 | 1.27 | 1.53 | 1 | This study |
| 12 | JLS170-19-2879 | 4 | M4 | 1.34 | 1.77 | 1 | This study |
| 13 | JLS170-19-296 | 4 | M4 | 1.56 | 1.73 | 1 | This study |
| 14 | JLS170-19-295 | 4 | M4 | 1.77 | 1.90 | 1 | This study |
| 15 | JLS170-19-1902 | 5 | M5 | 1.48 | 1.62 | 1 | This study |
| 16 | JLS170-19-2945 | 5 | M5 | 1.48 | 1.66 | 1 | This study |
| 17 | JLS160-19-1360 | 5 | M5 | 1.71 | 2.21 | 1 | This study |
| 18 | JLS160-19-591 | 5 | M5 | 1.69 | 1.97 | 1 | This study |
| 19 | JLS170-19-1375 | 5 | M5 | 1.76 | 2.04 | 1 | This study |
| 20 | JLS170-19-827 | 5 | M5 | 1.77 | 2.13 | 1 | This study |
| 21 | JLS170-19-1559 | 5 | M5 | 1.84 | 2.18 | 1 | This study |
| 22 | JB137-64 | 5 | M5 | 1.87 | 1.85 | 1 | This study |
| 23 | JLS160-19-611 | 5 | M5 | 1.88 | 2.30 | 1 | This study |
| 24 | LLS90-19-2 | 5 | M5 | 1.98 | 2.30 | 1 | This study |
| 25 | JLS82-19-399 | 5 | M5 | 2.14 | 2.03 | 1 | This study |
| 26 | JLS86-19-53 | 5 | M5 | 2.35 | 2.51 | 1 | This study |
| 27 | JLS160-19-612 | 6 | M6 | 1.75 | 2.06 | 1 | This study |
| 28 | JLS170-19-1484 | 6 | M6 | 2.28 | 2.22 | 1 | This study |
| 29 | JLS170-19-459 | 6 | M6 | 2.45 | 2.60 | 1 | This study |
| 30 | JLS170-19-779 | 6 | M6 | 2.44 | 2.32 | 1 | This study |
| 31 | JLS85-19-401 | 6 | M6 | 3.27 | 3.50 | 1 | This study |
| 32 | JLS170-19-78 | 6 | M6 | 3.38 | 3.51 | 1 | This study |
| 33 | JLS170-19-1482 | 6 | M6 | 3.37 | 3.52 | 1 | This study |
| 34 | JLS170-19-2819 | 6 | M6 | 3.49 | 3.38 | 1 | This study |
| 35 | JLS170-19-18533 | 6 | M6 | 3.46 | 3.32 | 1 | This study |
| 36 | JLS170-19-2978 | 7 | M7 | 2.42 | 2.15 | 1 | This study |
| 37 | JLS170-19-5901 | 7 | M7 | 3.13 | 3.52 | 1 | This study |
| 38 | JLS84.5-19-121 | 7 | M7 | 3.24 | 3.97 | 1 | This study |
| 39 | JLS84.5-19-473 | 7 | M7 | 3.53 | 2.73 | 1 | This study |
| 40 | JLS170-19-380 | 7 | M7 | 3.60 | 3.65 | 1 | This study |
| 41 | JLS170-19-650 | 7 | M7 | 3.62 | 3.91 | 1 | This study |
| 42 | JLS84-19-120 | 7 | M7 | 3.69 | 3.08 | 1 | This study |
| 43 | JLS170-18-463 | 8 | H | 4.26 | 4.30 | 1 | ★ |
| 44 | JLS160-19-1455 | 8 | H | 4.49 | 3.88 | 1 | This study |
| 45 | JLS162-19-142 | 8 | H | 4.50 | 4.29 | 1 | This study |
| 46 | JLS160-19-272 | 8 | H | 4.99 | 4.75 | 1 | This study |
| 47 | ST3 | 8 | H | 5.10 | 4.19 | 1 | This study |
| 48 | JLS160-19-403 | 8 | H | 511 | 4.52 | 1 | This study |
| 49 | JLS170-19-1446 | 8 | H | 6.09 | 5.11 | 1 | This study |
|  | | | | | | | |
| Serial number | **Henningsmoen's configuration in *Arthricocephalites xinzhaiheensis*** | | | | | | |
|  | Number | Number of thoracic segment | Stage | Length of trunk (μm) | Width of trunk (μm) | Inversion of cranidium | Data sources |
| 50 | JLS170-17-11 | 6 | M6 | 1.92 | 2.24 | no cranidium | ★★ |
| 51 | JLS85-16-712 | 6 | M6 | 2.55 | 2.35 | 0 | ★★ |
| 52 | JLS170-19-1377 | 6 | M6 | 3.33 | 3.53 | 1 | This study |
| 53 | JLS170-19-1768 | 7 | M7 | 2.79 | 2.87 | 0 | This study |
| 54 | JLS170-19-136 | 7 | M7 | 2.90 | 2.87 | no cranidium | This study |
| 55 | JB134-17-2 | 7 | M7 | 3.00 | 2.95 | no cranidium | ★★ |
| 56 | JLS85-17-4 | 7 | M7 | 3.36 | 2.43 | no cranidium | ★ |
| 57 | JLS170-19-1764 | 7 | M7 | 3.48 | 3.63 | no cranidium | This study |
| 58 | JLS170-19-944 | 7 | M7 | 3.38 | 3.05 | no cranidium | This study |
| 59 | JLS170-19-719 | 7 | M7 | 3.45 | 3.00 | no cranidium | This study |
| 60 | JLS170-19-1378 | 7 | M7 | 3.50 | 3.61 | no cranidium | This study |
| 61 | JLS170-17-9 | 7 | M7 | 3.50 | 2.95 | no cranidium | ★★ |
| 62 | JLS85-17-3 | 7 | M7 | 3.58 | 3.75 | 0 | ★★ |
| 63 | JLS170-19-14812 | 7 | M7 | 3.69 | 3.20 | no cranidium | This study |
| 64 | JLS170-18-3 | 7 | M7 | 3.65 | 3.08 | no cranidium | ★★ |
| 65 | JLS85-16-1 | 7 | M7 | 3.69 | 3.08 | 0 | ★★ |
| 66 | JLS170-19-1767 | 7 | M7 | 3.73 | 3.68 | no cranidium | This study |
| 67 | JLS85-17-1 | 7 | M7 | 3.70 | 3.01 | 0 | ★★ |
| 68 | JLS170-17-12 | 7 | M7 | 3.73 | 3.08 | no cranidium | ★★ |
| 69 | ST228-17-2 | 7 | M7 | 3.96 | 3.15 | no cranidium | ★★ |
| 70 | JLS85-17-2 | 7 | M7 | 4.31 | 3.13 | 0 | ★★ |
| 71 | JLS170-18-8 | 7 | M7 | 4.32 | 4.10 | no cranidium | ★★ |
| 72 | JLS170-19-3 | 7 | M7 | 4.46 | 4.88 | no cranidium | ★ |
| 73 | JLS170-18-9 | 7 | M7 | 4.79 | 4.58 | no cranidium | ★★ |
| 74 | JLS121-16-1 | 7 | M7 | 4.89 | 4.55 | no cranidium | ★★ |
| 75 | JLS170-19-737 | 7 | M7 | / | / | no cranidium | ★ |
| 76 | JLS170-19-1732 | 8 | H | 4.02 | 4.14 | no cranidium | This study |
| 77 | JLS84.5-19-692 | 8 | H | 4.44 | 3.21 | no cranidium | This study |
| 78 | JLS170-17-8 | 8 | H | 4.07 | 3.42 | no cranidium | ★ |
| 79 | JLS125-16-1 | 8 | H | 4.09 | 3.65 | no cranidium | ★ |
| 80 | JLS85-19-691 | 8 | H | 4.18 | 3.60 | no cranidium | This study |
| 81 | JLS170-19-220 | 8 | H | 4.23 | 3.64 | 1 | ★ |
| 82 | JLS160-19-996 | 8 | H | 4.36 | 4.11 | 1 | ★ |
| 83 | JLS170-18-11 | 8 | H | 4.27 | 3.48 | no cranidium | ★ |
| 84 | JLS170-19-14810 | 8 | H | 4.38 | 3.34 | no cranidium | This study |
| 85 | JLS170-17-7 | 8 | H | 4.38 | 3.18 | no cranidium | ★ |
| 86 | JLS170-19-128 | 8 | H | 4.32 | 4.27 | no cranidium | ★ |
| 87 | JLS170-16-61 | 8 | H | 4.38 | 4.01 | no cranidium | ★ |
| 88 | JLS170-19-943 | 8 | H | 4.61 | 4.07 | no cranidium | This study |
| 89 | JLS170-17-10 | 8 | H | 4.52 | 4.08 | no cranidium | ★ |
| 90 | JLS170-19-41 | 8 | H | 4.51 | 3.82 | 0 | ★ |
| 91 | JLS170-17-2 | 8 | H | 4.53 | 4.05 | 1 | ★ |
| 92 | JLS85-16-699 | 8 | H | 4.64 | 4.03 | no cranidium | ★ |
| 93 | JLS164-16-2 | 8 | H | 5.31 | 5.37 | no cranidium | ★ |
| 94 | JLS85-19-187 | 8 | H | 4.72 | 4.59 | no cranidium | This study |
| 95 | JLS170-17-1 | 8 | H | 4.80 | 4.04 | 1 | ★ |
| 96 | JLS170-18-4 | 8 | H | 4.83 | 3.83 | no cranidium | ★ |
| 97 | JLS84.5-19-186 | 8 | H | 5.00 | 4.06 | no cranidium | This study |
| 98 | JLS170-18-1 | 8 | H | 5.16 | 3.86 | no cranidium | ★ |
| 99 | JLS160-17-3 | 8 | H | 5.29 | 4.60 | no cranidium | ★ |
| 100 | JLS170-17-6 | 8 | H | 5.37 | 5.15 | no cranidium | ★ |
| 101 | JLS170-18-2 | 8 | H | 5.37 | 4.66 | no cranidium | ★ |
| 102 | JLS170-19-1540 | 8 | H | 5.45 | 3.90 | no cranidium | This study |
| 103 | JLS170-19-833 | 8 | H | 5.47 | 5.20 | no cranidium | This study |
| 104 | JLS85-17-6 | 8 | H | 5.41 | 4.01 | no cranidium | ★ |
| 105 | JLS170-19-98 | 8 | H | 5.23 | 4.49 | no cranidium | ★ |
| 106 | JLS85-17-7 | 8 | H | 5.51 | 3.81 | no cranidium | ★ |
| 107 | JLS170-18-5 | 8 | H | 5.53 | 4.49 | no cranidium | ★ |
| 108 | JLS85-17-8 | 8 | H | 5.60 | 4.14 | no cranidium | ★ |
| 109 | JLS160-17-4 | 8 | H | 5.57 | 5.15 | no cranidium | ★ |
| 110 | JLS87-16-1 | 8 | H | 5.65 | 5.60 | no cranidium | ★ |
| 111 | JLS160-17-1 | 8 | H | 5.74 | 4.47 | no cranidium | ★ |
| 112 | JLS85-16-314 | 8 | H | 5.84 | 5.89 | no cranidium | ★ |
| 113 | JLS85-19-188 | 8 | H | 6.02 | 3.99 | 0 | This study |
| 114 | JLS170-16-63 | 8 | H | 5.99 | 5.05 | no cranidium | ★ |
| 115 | JLS160-16-211 | 8 | H | 6.00 | 5.32 | no cranidium | ★ |
| 116 | JLS160-17-2 | 8 | H | 6.20 | 5.30 | no cranidium | ★ |
| 117 | ST317.4-19-1 | 8 | H | 6.12 | 5.44 | no cranidium | This study |
| 118 | JLS164-16-1 | 8 | H | 6.14 | 5.60 | no cranidium | ★ |
| 119 | JLS85-16-62 | 8 | H | 6.40 | 6.54 | no cranidium | ★ |
| 120 | JLS170-16-3 | 8 | H | 6.33 | 5.21 | no cranidium | ★ |
| 121 | JLS85-17-5 | 8 | H | 6.30 | 4.48 | no cranidium | ★ |
| 122 | JLS85-19-185 | 8 | H | 6.38 | 4.71 | no cranidium | This study |
| 123 | JLS160-19-1300 | 8 | H | 6.49 | 5.64 | no cranidium | ★ |
| 124 | JLS170-17-5 | 8 | H | 6.59 | 5.08 | no cranidium | ★ |
| 125 | JLS85-16-91 | 8 | H | 7.05 | 5.83 | no cranidium | ★ |
| 126 | JLS160-19-1006 | 8 | H | 7.34 | 6.24 | no cranidium | ★ |
| 127 | ST295-17-1 | 8 | H | 9.34 | 7.87 | no cranidium | ★ |
| 128 | JLS84.5-19-189 | 8 | H | / | / | no cranidium | This study |
| 129 | JLS85-16-63 | 8 | H | / | / | no cranidium | ★ |
| 130 | JLS170-16-1 | 8 | H | / | / | 1 | ★ |
| 131 | JLS160-19-1272 | 8 | H | / | / | no cranidium | ★ |

References

Wang YF, Peng J, Wang QJ, Wen RQ, Zhang H, Du GY, Shao YB. 2021 Moulting in the Cambrian oryctocephalid trilobite Arthricocephalites xinzhaiheensis from Guizhou Province, South China. Lethaia 54, 211–228. (doi:10.1111/let.12398)
